# Clinical features and outcomes of pseudolithiasis induced by ceftriaxone in Chinese children: a single-center observational study

**DOI:** 10.3389/fped.2025.1527014

**Published:** 2025-01-17

**Authors:** Chen Jin, Wen-Li Xiu, Yao Liu, Xi-Wei Hao, Qian Dong

**Affiliations:** ^1^ Department of Pediatric Surgery, The Affiliated Hospital of Qingdao University, Qingdao, Shandong, China; ^2^ Shandong Provincial Key Laboratory of Digital Medicine and Computer-Assisted Surgery, Qingdao University, Qingdao, Shandong, China

**Keywords:** ceftriaxone, gallbladder pseudolithiasis, pediatric infectious diseases, ultrasound imaging, serum biomarkers

## Abstract

**Introduction:**

Ceftriaxone (CTX) is widely used in pediatric infectious disease treatment, although the diagnostic and therapeutic management of CTX-induced gallbladder pseudolithiasis (PL) remains challenging. In this study, we investigated the occurrence, clinical features, and management of CTX-induced PL in children.

**Methods:**

A retrospective case-control study was conducted on 185 pediatric patients receiving CTX at a single center. Data on treatment regimens, gallbladder imaging findings, and serum biochemical parameters post-CTX therapy were analyzed. Patients were classified into PL (*n* = 34) and non-PL (*n* = 151) groups based on imaging findings.

**Results:**

PL was diagnosed in 18.4% of patients treated with CTX, primarily through ultrasound, which revealed hyperechoic material within the gallbladder. Compared with the non-PL group, patients with PL were older and taller, with no significant differences in CTX dosage (*p* = 0.915). Patients with PL also had higher rates of digestive and neurological infections (both *p* < 0.001). Serum analysis revealed distinct liver and kidney function markers in the PL group, including lower levels of total bile acids, adenosine deaminase, and lactate dehydrogenase, and higher creatinine levels (all *p* < 0.05). Discontinuation of CTX led to symptom resolution in most cases, and all cases of PL resolved within three months.

**Conclusions:**

The occurrence of PL is not significantly related to CTX dosage. Furthermore, the rate of CTX metabolism and excretion may play a key role in PL development. Overall, the findings demonstrate that ultrasound is an effective tool for monitoring the development of PL in children receiving CTX and that discontinuation of CTX could be an effective treatment for PL.

## 1 Introduction

Ceftriaxone (CTX) is a third-generation cephalosporin that is widely used to treat respiratory, central nervous system, and gastrointestinal infections in children. Approximately 40% of this drug is excreted via the hepatobiliary system, where it precipitates with calcium ions to form gallbladder deposits. This leads to a condition known as pseudolithiasis (PL) (1). Unlike true gallstones, PL is typically reversible and resolves spontaneously (2, 3).

The development of PL is associated with factors such as bile acid secretion, CTX concentration, and gallbladder motility (4). Reduced gallbladder contractility is commonly observed in critically ill patients receiving parenteral nutrition and is a significant risk factor (1, 5). Additionally, CTX may directly inhibit gallbladder contractions and intestinal motility, further increasing the risk of PL (6).

The relationship between CTX dosage and PL remains controversial. While some studies suggest that high-dose CTX (>100 mg/kg) increases the risk of PL, others report no significant associations (7, 8). Similarly, conflicting findings exist regarding the role of treatment duration in the development of PL (9, 10). Although numerous studies have investigated the etiology and factors influencing CTX-induced PL, comprehensive research on its clinical features, diagnostic methods, and management strategies remains limited.

PL is common in pediatric CTX patients, often causing overlooked abdominal symptoms, with improper management leading to subsequent health issues. In this study, we aimed to retrospectively analyze pediatric patients receiving CTX treatment to identify CTX-induced PL. By comparing imaging findings and liver and kidney functions between PL and non-PL patients, we sought to enhance our understanding of PL pathogenesis and refine approaches to its clinical management.

## 2 Materials and methods

### 2.1 Participants

**Figure 1:**
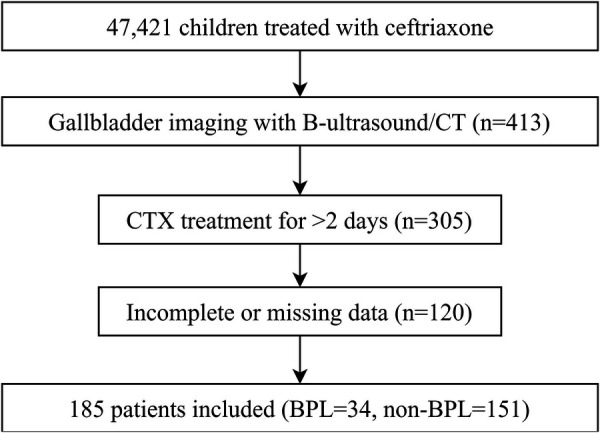
Flowchart of patient selection.

This retrospective case-control study was conducted at the Affiliated Hospital of Qingdao University between April 2003 and December 2023. Pediatric patients treated with CTX were identified by screening their medical records. The study protocol was approved by the Institutional Ethics Committee (Ethics Number: QYFYWZLL27865), and written informed consent was obtained from parents or guardians. Figure 1 depicts a flowchart detailing patient selection.

Patients aged 1–17 years who received CTX treatment were included in the study. The inclusion criterion was gallbladder imaging [ultrasound or computed tomography (CT)] within two weeks of CTX therapy. Exclusion criteria included a CTX treatment duration of <2 days, a history of gallbladder disease, use of medications associated with gallstone formation, end-stage organ failure, malignancy, or reliance on parenteral nutrition.

In this study, all enrolled pediatric patients underwent gallbladder imaging examinations (ultrasound or CT) as part of the primary disease screening or in response to the onset of new abdominal symptoms. The diagnosis of PL was established based on CTX administration and specific gallbladder imaging findings (11, 12), which included: (1) administration of at least one prescribed dose of CTX, (2) identification of movable abnormal echogenicity within the gallbladder on ultrasound or abnormal density within the gallbladder on CT, and (#) disappearance of gallbladder stones on ultrasound or CT within 3 months after cessation of CTX sodium.

### 2.2 Data collection

Patient demographics, medical histories, treatment regimens, liver and kidney function tests, and imaging results were retrieved from electronic medical records. Liver and kidney function tests were performed after fasting using a Hitachi 7,600 analyzer. Gallbladder imaging was performed using ultrasonography (Philips IU22) or CT (Somatom Definition). Ultrasound evaluations followed a 12-h fasting period and were performed in multiple positions to detect echogenic material within the gallbladder.

### 2.3 Statistical analysis

Categorical variables are presented as frequencies and percentages, and comparisons were conducted using the chi-square test. The normality of continuous variables was assessed using the Kolmogorov–Smirnov test. Normally distributed data are expressed as mean ± standard deviation and were compared using the *t*-test. Non-normally distributed data are expressed as medians and interquartile ranges (IQRs), and comparisons were made using the Mann–Whitney *U*-test. Statistical analysis was performed using R statistical software (version 4.3.2), and a *p*-value <0.05 was considered statistically significant.

## 3 Results

### 3.1 Diagnosis

**Figure 2:**
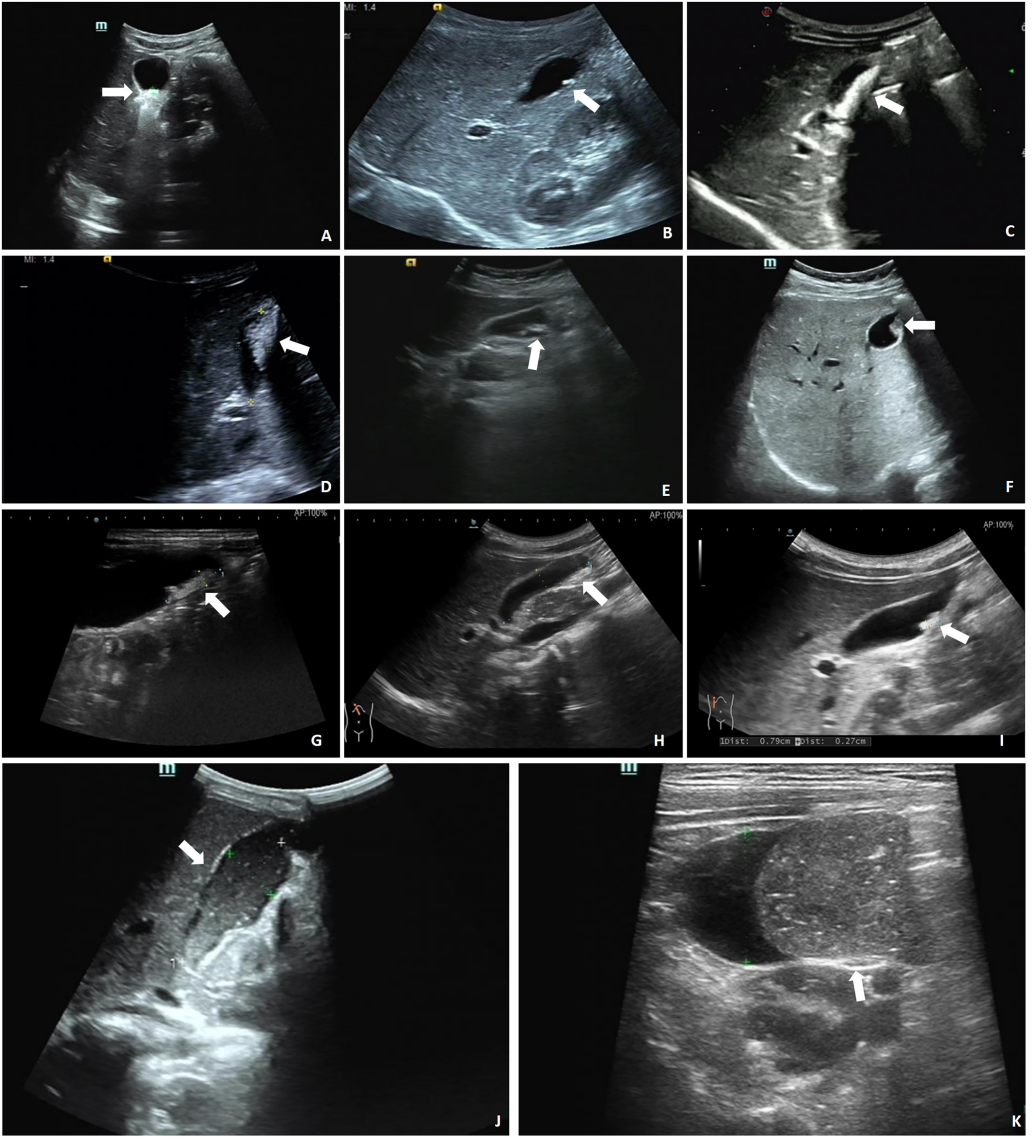
Ultrasound in the gallbladder. **(A)** 0.8 × 0.4 cm strong echo. **(B)** 0.6 × 0.2 cm strong echo. **(C)** Sand-like echoes, 2.6 × 0.7 cm. **(D)** Sand-like echoes, 2.2 × 1.1 cm. **(E)** Sand-like echoes, 2.2 × 0.4 cm. **(F)** Accumulated strong echoes, 1.2 × 0.7 cm and 1.0 × 0.5 cm. **(G)** 1.6 × 0.4 cm strong echo. **(H)** 1.9 × 0.5 cm strong echo. **(I)** 0.8 × 0.3 cm strong echo. **(J)** 5.6 × 2.1 cm low echo. **(K)** 5.6 × 1.9 cm low echo. Arrows indicate strong/low echoes in the gallbladder.

**Figure 3:**
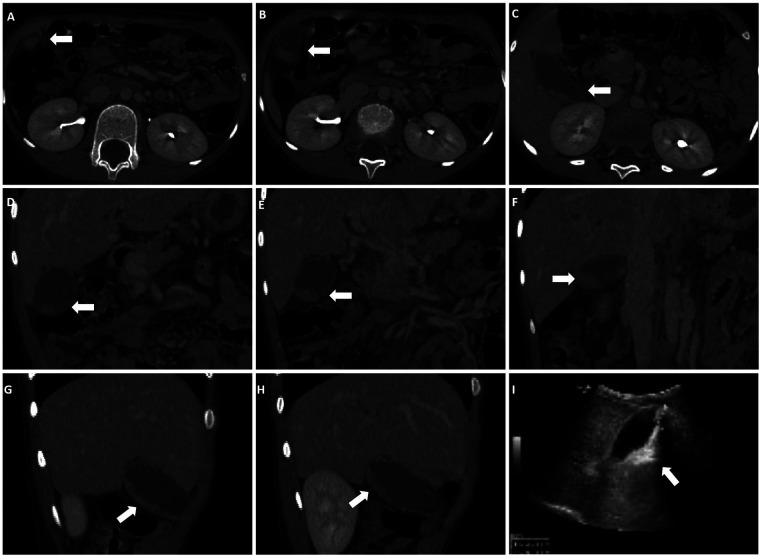
Both CT and ultrasound examinations reveal PL. **(A–C)** Cross-sectional CT images. **(D–F)** Coronal CT images. **(G,H)** Sagittal CT images. **(I)** ultrasound shows a 3.1 × 1.0 cm strong echo within the gallbladder lumen, accompanied by acoustic shadowing. Arrows indicate the presence of high-density shadows and strong echoes within the gallbladder.

A total of 185 pediatric patients were included in this study. All patients underwent abdominal imaging 3–5 days after CTX administration, which revealed that 34 (18.38%) patients were diagnosed with PL. The patients were classified into two groups: PL (*n* = 34) and non-PL (*n* = 151). Among the diagnosed PL cases, 24 (70.59%) were confirmed by ultrasound, which showed various morphologies. Of these, 23 (67.65%) cases exhibited strong echogenicity within the gallbladder (Figures 2A–I), while one (2.94%) case exhibited low echogenicity (Figures 2J,K). CT scans confirmed six (17.65%) cases, revealing high-density shadows, while four (11.76%) cases were diagnosed using both CT and ultrasound (Figure 3).

Among the patients with PL, 14 (47.18%) presented with abdominal symptoms, including pain (5 cases), vomiting (2 cases), abdominal pain with vomiting (6 cases), and abdominal pain with nausea (1 case). The remaining 20 (58.8%) patients were asymptomatic and diagnosed incidentally during the investigation of the primary disease.

Among the 46 children under 3 years of age, four had PL, with three (75%) presenting with abdominal symptoms, including two cases of vomiting and one case of abdominal pain with vomiting. Of the 42 children without PL, three (7.14%) had abdominal symptoms, mainly vomiting. The incidence of abdominal symptoms was significantly higher in the PL group. Among children older than three years, 30 of 139 (21.58%) had PL, and 11 (36.67%) exhibited abdominal symptoms. Among the 109 children without PL, 7 (6.42%) had abdominal symptoms. Abdominal symptoms were more common in patients with PL, especially in those under three years of age.

### 3.2 Patient characteristics

The basic characteristics and primary disease conditions of both patient groups are summarized in Table 1. Compared to patients without PL, those with PL were older (*p* = 0.025) and taller (*p* = 0.043). Although both groups primarily presented with respiratory infections, patients with PL had a significantly higher incidence of infections affecting the digestive and nervous systems (both *p* < 0.05). No significant differences were observed between the two groups in terms of sex, weight, or body mass index (BMI) (*p* < 0.05).

**Table 1:** Comparison of baseline characteristics between Two groups of pediatric patients.

Variable	PL (+), *n* = 34	PL (−), *n* = 151	X^2^/*U*	*P*
Sex, male, *n* (%)	19 (55.9)	61 (40.4)	0.499^a^	0.480
Age, months	80 (53.22–130.87)	62.63 (39.47–101.27)	1,321.5^b^	0.021
Height, cm	121 (110–145)	111 (98–131)	1,383^b^	0.043
Weight, kg	22 (17.5–37.75)	19 (14–27)	1,400^b^	0.052
BMI, kg/cm^2^	16.41 (14.96–18.84)	16.44 (14.47–17.99)	1,771^b^	0.843
Infection, *n* (%)				
Respiratory system	17 (50.0)	76 (50.3)	0.001^a^	0.972
Urinary system	0 (0)	6 (4.0)	1.396^a^	0.237
Digestive system	15 (44.1)	25 (16.6)	12.439^a^	<0.001
Nervous system	6 (17.6)	4 (2.6)	12.208^a^	<0.001

^a^ Chi-square test.

^b^ Mann–Whitney *U*-test.

### 3.3 Examination, treatment, and prognosis

Medication dosages did not differ between the two groups. The duration of CTX treatment was shorter in PL patients (median = 4 days, IQR = 3–5.5 days) compared to the non-PL group (median = 6 days, IQR = 4–8 days) (*p* = 0.035). Blood tests for liver and kidney function after medication revealed that total bile acid (TBA), adenosine deaminase (ADA), lactate dehydrogenase (LDH), α-L-fucosidase (AFU), aspartate aminotransferase (AST), alanine aminotransferase (ALT), and blood urea-to-creatinine ratio (BUN/cre) were lower in the PL group compared to the non-PL group, while creatinine (cre) levels were higher (*p* < 0.05) (Table 2). Blood tests, as well as liver and kidney function tests were completed within six days of CTX administration in both the PL and non-PL groups (*p* = 0.3918 and *p* = 0.4555, respectively). Other liver and kidney function tests showed no significant differences between the groups.

**Table 2:** Comparison of ceftriaxone (CTX) usage and hepatic-renal function parameters post-medication.

Variable	PL (+), *n* = 34	PL (−), *n* = 151	*U*-value	*p*-value
Dose, mg/kg	77.27 (56.6–85.785)	76.19 (63.49–80)	1,836.5	0.915
Total dose, mg	282.74 (208.7–472.68)	400 (241.22–583.31)	2,982	0.1417
Duration, days	4 (3–5.5)	6 (4–8)	2,257.5	0.035
TBA, umol/L	1.8 (0.85–3.79)	3.9 (2.18–7)	2,664	0.000
ADA, U/L	14 (12–21)	19 (15–27)	2,442.5	0.003
LDH, U/L	195 (180.5–268)	256 (207–313)	2,443.5	0.003
AFU, U/L	22 (19.5–23)	25 (21–34)	2,439	0.003
ALT, U/L	13.8 (9.4–18)	18 (12–29)	2,345	0.012
AST, U/L	24 (17–32.5)	28 (22–39)	2,307	0.020
Cre, umol/L	57 (51.85–66.3)	50.7 (39–62)	1,322.5	0.021
BUN/cre	11.02 (7.7–19.29)	14.75 (10.19–23.56)	2,245.5	0.042

TBA, total bile acids; ADA, adenosine deaminase; LDH, lactate dehydrogenase; AFU, α-L-fucosidase; ALT, glutamate pyruvate transaminase; AST, glutamic oxaloacetic transaminase; Cre, creatinine; BUN, blood urea nitrogen.

In a previous study (11), hypoalbuminemia was identified as a risk factor for PL. In the PL group, eight (23.53%) patients had hypoalbuminemia (serum albumin <30 g/L) compared with six (3.97%) patients in the non-PL group. However, albumin levels did not differ significantly between the groups (*P* = 0.1417).

Upon diagnosis of PL, patients discontinued CTX treatment. Among the patients with abdominal symptoms, 11 (78.57%) had symptom resolution within three days after discontinuation of CTX, while two (14.29%) still had symptoms after three days but improved with ursodeoxycholic acid treatment. One patient experienced exacerbation of symptoms and developed acute cholecystitis. Treatment with magnesium isoglycyrrhizinate relieved abdominal pain, which completely resolved after four days. All children were followed up within three months, and gallbladder ultrasonography and CT revealed disappearance of the stones. Patients in the non-PL group completed the CTX treatment cycle according to clinical indications.

After discontinuation of CTX, 22 (64.71%) patients completed their treatment, while 12 (35.29%) switched to alternative antibiotics to continue anti-infection therapy. This group included six patients with new-onset abdominal symptoms and six without. Alternative antibiotics included cefuroxime sodium, piperacillin-tazobactam sodium, linezolid, mezlocillin sodium-sulbactam sodium, latamoxef sodium, ceftazidime, and cefdinir. All infections were treated effectively.

## 4 Discussion

In clinical practice, the understanding of CTX-induced PL is limited, and research on its diagnosis and clinical manifestations is sparse. In this study, 34 (18.38%) cases of PL were identified and primarily assessed using ultrasound, which aligns with the reported incidence range of 17%–26% in previous studies (8, 12, 13). PL is typically characterized by strong echoes within the gallbladder, with nearly half of the patients exhibiting abdominal pain. Compared to patients without PL, those with PL were older, taller, and had a higher incidence of infections in the digestive and nervous systems. Hypoalbuminemia was more common in patients with PL, along with notable differences in liver and kidney function parameters. After discontinuation of CTX treatment, abdominal symptoms resolved in most patients, with only three patients requiring pharmacological intervention. Follow-up examinations confirmed the complete resolution of PL in all patients.

Although many patients remain asymptomatic, abdominal pain, nausea, and vomiting are common symptoms of PL (14). Our study found that pediatric PL typically presents with hypoechoic or hyperechoic gallbladder sludge and echogenic stones on ultrasonography, which is consistent with findings in adults (13, 15). These findings underscore the critical role of imaging in diagnosing PL. Furthermore, the presence of these features could help distinguish PL from other gallbladder conditions, providing valuable insights into disease progression and potential complications.

The higher incidence of digestive and neurological infections in patients with PL may be linked to reduced food intake, a known risk factor. Patients on total parenteral nutrition or those unable to eat lack the gallbladder contraction stimulus, which increases the risk of PL (16–18). Maintaining a proper diet during CTX treatment could help reduce the risk of PL in vulnerable pediatric patients.

An increased CTX dose, defined as exceeding 2 g/day in adults or 100 mg/kg/day in pediatric patients, is considered a risk factor for PL (7); however, the relationship remains inconclusive. In our study, all patients received CTX doses not exceeding 2 g/day. The median dose for the PL group was 77.27 mg/kg (range: 56.6–85.8 mg/kg), while for the non-PL group, it was 76.19 mg/kg (range: 63.49–80 mg/kg). In addition to dosage, pharmacokinetic variability (such as individual metabolic rates) and genetic factors (such as variations in drug-metabolizing enzymes) are also associated with drug excretion levels. Therefore, the CTX metabolism rate, rather than dosage, may play a key role in PL development.

Further analysis revealed that patients in the PL group were older and taller than those in the non-PL group. Additionally, patients with PL exhibited lower levels of TBA, ADA, LDH, laminin, ALT, AST, and BUN/cre, but higher creatinine levels. As children grow, their liver matures, improving hepatic metabolic capacity, which is reflected in significant changes in liver function markers. This supports the hypothesis that increased CTX metabolism may be associated with PL formation.

TBA is synthesized in the liver, aids in fat digestion through bile secretion, and re-enters the liver via enterohepatic circulation (19). When liver function declines, as in cirrhosis or hepatitis, TBA synthesis increases, leading to elevated serum levels. In contrast, optimal liver function reduces TBA synthesis and lowers serum TBA levels. Additionally, elevated CTX excretion can inhibit bile acid secretion, further reducing serum TBA levels (4). Thus, the decrease in TBA may result from the competitive inhibition of CTX hepatic excretion.

CTX may also affect liver function. Kazuhiko et al. found that high-dose CTX was associated with a higher incidence of liver damage than were standard doses (20). Carmen et al. reported a higher risk of liver injury in children treated with CTX (odds ratio: 26.70, 95% confidence interval: 12.09–58.96) (21), though the dosage was unspecified. Rakhee et al. found no significant association between CTX dosage and elevated liver enzyme levels in adults (22). Some studies suggest that CTX may reduce hepatic oxidative stress and protect the liver (23, 24). One study by Elham et al. demonstrated the protective effects of CTX against liver function and oxidative stress in a mouse model (25). In conclusion, CTX may have both damaging and protective effects on liver function in children. The decreased levels of TBA, ADA, LDH, AFU, ALT, and AST in patients with PL may be attributed to the effects of CTX or improved liver function with age.

This study has several limitations, including the small sample size. Infants under 1 year of age, who had a higher incidence of PL, were not included, and further studies focusing on this group are warranted.

## 5 Conclusions

We identified 34 cases (18.38%) of CTX-induced pediatric PL, with ultrasonography playing a key role in diagnosis. Abdominal pain was a common symptom, but many patients were asymptomatic. The findings suggest that dietary intake and CTX metabolism, rather than dosage, may be critical factors in PL development. Discontinuing CTX medication upon PL diagnosis was effective in resolving symptoms in most cases. Symptomatic treatments, including litholytic, choleretic, and hepatoprotective therapies, may be considered if PL symptoms persist. Additionally, diligent follow-up is essential to confirm the diagnosis and exclude pediatric cholelithiasis.

## Data Availability

The raw data supporting the conclusions of this article will be made available by the authors, without undue reservation.
